# A Functional Clock Within the Main Morning and Evening Neurons of *D. melanogaster* Is Not Sufficient for Wild-Type Locomotor Activity Under Changing Day Length

**DOI:** 10.3389/fphys.2020.00229

**Published:** 2020-03-26

**Authors:** Pamela Menegazzi, Katharina Beer, Verena Grebler, Matthias Schlichting, Frank K. Schubert, Charlotte Helfrich-Förster

**Affiliations:** Neurobiology and Genetics, Theodor-Boveri Institute, Biocenter, University of Würzburg, Würzburg, Germany

**Keywords:** entrainment (light), two-oscillator model, photoperiod alterations, *drosophila melanogaster* meigen, clock neurons

## Abstract

A major challenge for all organisms that live in temperate and subpolar regions is to adapt physiology and activity to different photoperiods. A long-standing model assumes that there are morning (M) and evening (E) oscillators with different photoreceptive properties that couple to dawn and dusk, respectively, and by this way adjust activity to the different photoperiods. In the fruit fly *Drosophila melanogaster*, M and E oscillators have been localized to specific circadian clock neurons in the brain. Here, we investigate under different photoperiods the activity pattern of flies expressing the clock protein PERIOD (PER) only in subsets of M and E oscillators. We found that all fly lines that expressed PER only in subsets of the clock neurons had difficulties to track the morning and evening in a wild-type manner. The lack of the E oscillators advanced M activity under short days, whereas the lack of the M oscillators delayed E activity under the same conditions. In addition, we found that flies expressing PER only in subsets of clock neurons showed higher activity levels at certain times of day or night, suggesting that M and E clock neurons might inhibit activity at specific moments throughout the 24 h. Altogether, we show that the proper interaction between all clock cells is important for adapting the flies’ activity to different photoperiods and discuss our findings in the light of the current literature.

## Introduction

Endogenous clocks that tick with an ∼24 h period control circadian rhythms. They entrain to the 24 h cycles of the earth via external Zeitgebers, the strongest of which is light. Since activity must occur at the most favorable time of the day, the rest–activity rhythm is one of the most tightly clock-controlled behaviors. In natural conditions, many animal species display bimodal rest–activity profiles with pronounced morning (M) and evening (E) activity bouts, and little activity during the middle of the day or night (Aschoff, 1966; Saunders, 2002; Dunlap et al., 2004). In long summer days, M activity occurs earlier and E activity later, helping the animals to avoid the midday heat by being active mainly in the morning and evening. Such an adaptation is especially important for small insects such as fruit flies that are in danger of desiccation (Hamblen-Coyle et al., 1992; Majercak et al., 1999; Bywalez et al., 2012). This behavior occurs also in the laboratory under light–dark (LD) cycles but constant temperatures showing that light is the major cue that drives these changes (Rieger et al., 2003, 2007, 2012; Shafer et al., 2004; Menegazzi et al., 2017; Schlichting et al., 2019b).

The long-standing two-oscillator model of Pittendrigh and Daan (1976), originally developed for mammals, explains the described seasonal adaptations by assuming an M oscillator that shortens its period and an E oscillator that lengthens its period when exposed to light for an extended time. The cellular basis of the two oscillators has been described first in the fruit fly: M and E oscillators are located in distinct groups of circadian clock neurons – the so-called M and E neurons (Grima et al., 2004; Stoleru et al., 2004; Rieger et al., 2006).

**FIGURE 1:**
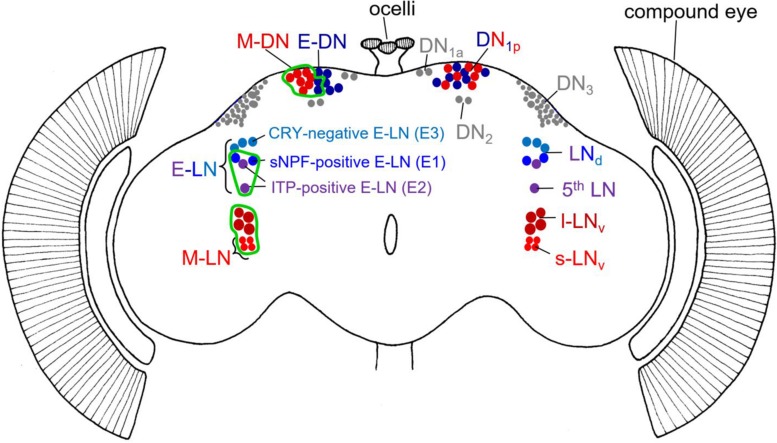
Schematic representation of the different clock neurons in the *Drosophila* brain. The right brain hemisphere depicts the traditional division in different clock neurons including their classification in morning (M) and evening (E) neurons in reddish and bluish colors, respectively. Clock neurons that cannot be unequivocally assigned as M or E neurons are shown in gray. Note that the DN_1__p_ consist of a mixture of M and E neurons. The left-brain hemisphere depicts the M and E neurons in more detail and indicates in which specific neurons we rescued PER in *per*^0^ mutants (green edging). Due to limited gal4-drivers, we were not always able to restrict PER to only M or E neurons. For example, our M-DN driver (*Clk4.1M-gal4*) included also ∼2 DN_1__p_ that belong to the E-DN. Note that we clustered M- and E-DN in the left brain hemisphere to indicate the expression of the *Clk4.1M-gal4* driver line. In the case of the M-LN, only the s-LN_v_ are bonafide M-oscillators, but by using the *Pdf-gal4* driver, we rescued PER also in the l-LN_v_. In the case of E-LN, we rescued PER in the sNPF-positive (E1) and ITP-positive (E2) neurons (using the *PDF-gal80 Mai179-gal4* driver). The Cryptochrome (CRY)-negative neurons (E3) are not included.

The *Drosophila* brain clock consists of ∼150 neurons that express the PERIOD (PER) protein and are divided into different clusters of lateral and dorsal neurons (LN and DN) (Figure 1). All clock neurons form an interconnected neuronal network that has been partially morphologically and functionally dissected (Rieger et al., 2006; Shafer et al., 2006; Helfrich-Förster et al., 2007; Yao and Shafer, 2014; Schubert et al., 2018).

As defined in original work, the M neurons consist of a ventral group of the lateral clock neurons – the four PDF-positive small ventral lateral neurons (s-LN_v_) – whereas the E cells are composed of the dorsal group of the LNs, the LN_d_ (Grima et al., 2004; Stoleru et al., 2004). Later work showed that the so-called 5th s-LN_v_ also behaves as an E oscillator, whereas only three of the six LN_d_ lengthen their period in response to light and thus work as bonafide E oscillators (Rieger et al., 2006). These three E-LN_d_ are most likely identical with the three cryptochrome (CRY)-expressing LN_d_ cells (Picot et al., 2007). Work that is more recent showed that the three CRY-positive E-LN_d_ can be further divided into two short neuropeptide F (s-NPF)-expressing neurons and one ion transporter peptide (ITP)-expressing cell (Figure 1; Johard et al., 2009). Most interestingly, ITP is also present in the 5th s-LN_v_, and this cell turned out to be morphologically very similar to the ITP-positive E-LN_d_ cell and not with the PDF-positive M s-LN_v_ cells, suggesting that the 5th s-LN_v_ belongs to the E-LN_d_ neurons (Schubert et al., 2018). Even functionally, the two ITP-positive E neurons are closely related (Yao and Shafer, 2014). Therefore, to avoid confusion with the LN_v_ M neurons, we proposed to refer to the 5th s-LN_v_ simply as 5th LN (see Figure 1). As depicted in Figure 1, Yao and Shafer (2014) classified the E cells into three groups, E1, E2, and E3. E1 corresponds to the s-NPF-positive E-LN, E2 corresponds to the ITP-expressing E-LN, and E3 corresponds to the CRY-negative E-LN (Figure 1). Depending on the environmental conditions, the three groups of E-LN appear to behave differently (Rieger et al., 2009; Yoshii et al., 2012; Yao and Shafer, 2014). There are also indications that the ∼15 DN_1__p_ dorsal neurons consist of M and E oscillators. Half of them express CRY and the simplest view is that the CRY-negative DN_1__p_ are E neurons while the CRY-positive DN_1__p_ are M neurons (Murad et al., 2007; Zhang Y. et al., 2010; Yoshii et al., 2012). In the following, we call these neurons E-DN and M-DN, respectively (Figure 1).

Consistent with the two-oscillator model, *Drosophila*’s M and E neurons respond differently to light, with M cells shortening their period and some E cells lengthening their period when the flies are exposed to constant light conditions (Rieger et al., 2006; Yoshii et al., 2012). The M neurons advance their phase under dim constant light and light moonlight cycles, whereas the E neurons delay their phase, thereby lengthening the time between M and E activity peaks (Bachleitner et al., 2007).

To understand the contribution of the different clock neurons to rhythmic behavior, a UAS-*period* transgene was generated in order to rescue *per* expression with the help of specific *gal4-drivers* in subsets of M or E neurons of *per*^0^ mutant flies (Grima et al., 2004). As expected, these flies show differences in the appearance of M and E activity bouts when recorded under LD cycles with 12 h of light and 12 h of darkness (LD 12:12) (Grima et al., 2004; Picot et al., 2007; Zhang L. et al., 2010; Zhang Y. et al., 2010). Nevertheless, so far, the activity of these flies has not been recorded under different photoperiods. If the original Pittendrigh-Daan two-oscillator model is valid, and M and E oscillators control M and E activity in an autonomous manner, the morning activity of flies with functional M oscillators should track lights-on even in the absence of the E oscillators. Vice versa, the evening activity of flies with functional E oscillators should track lights-off even in the absence of the M oscillators. Here, we tested this hypothesis and found that the situation is more complex. Our results are in line with the findings of several other groups that manipulated the molecular clocks in M and E oscillators in different ways (Picot et al., 2007; Stoleru et al., 2007; Zhang L. et al., 2010; Zhang Y. et al., 2010; Guo et al., 2014, 2016, 2018; Chatterjee et al., 2018; Schlichting et al., 2019b). In the end, we show that the original M and E oscillator model is too simple to explain all findings.

## Materials and Methods

### Fly Strains

To restrict PER expression to specific M or E clock neurons, we started from arrhythmic *per*^0^ mutants and rescued PER with the help of the UAS-Gal4 system in subsets of the clock neurons as done previously (Grima et al., 2004; Picot et al., 2007; Zhang L. et al., 2010; Zhang Y. et al., 2010). The neurons to which PER is finally restricted are shown in Figure 1. In the following, we will describe the employed lines and the crosses that yielded the experimental and control animals.

*Pdf-gal4/* + flies, *Mai179-gal4/* + flies, *tim-gal4/* + flies, *Clk4.1 M-gal4* flies, *per*^0^; *uas-per16*, and *Pdf-gal80* were described previously (Renn et al., 1999; Kaneko and Hall, 2000; Siegmund and Korge, 2001; Grima et al., 2004; Stoleru et al., 2004; Zhang L. et al., 2010).

In order to get flies with PER only in the M-LN cells, we crossed female *per*^0^;*uas-per16* to male *Pdf-gal4/* + flies and selected the male offspring to obtain *per^0^;Pdf-gal4/* +;*uas-per16/* + flies. To get flies with PER in the majority of the LN (M- and E-LN flies), we crossed female *per^0^;uas-per16* to male *Mai179-gal4/* + flies and obtained male *per^0^;Mai179-gal4/* +;*uas-per16/* + flies. We also generated a stable per*^0^;Mai179-gal4*;*uas-per16* that was crossed to the *Pdf-gal80* strain to obtain w *per^0^;Mai179-gal4/Pdf-gal80;uas-per16/* + males that express PER only in the E-LN. These flies express PER in the sNPF-positive and ITP-positive E-LN (E1 and E2 cells) (Yao and Shafer, 2014; Schubert et al., 2018). Male flies that express PER in 8–10 DN_1__p_ cells were obtained by crossing male *Clk4.1 M-gal4* flies with female *per*^0^;*uas-per16* flies. The genotype of these flies is *per^0^;Clk4.1 M-gal4/uas-per16*. These flies express PER in all CRY-positive DN_1__p_ cells, which are regarded as M cells (Yoshii et al., 2012). Therefore, for simplicity, we call these flies M-DN flies, in spite of the fact that they express PER additionally also in some CRY-negative neurons (Chatterjee et al., 2018). *Clk4.1 M-gal4* males were also crossed to per*^0^;Mai179-gal4*;*uas-per16* females to obtain per*^0^;Mai179-gal4/* +; *Clk4.1 M-gal4*/*uas-per16* that express PER in the M-LN, E-LN, and M-DN. As positive control flies, we used *per^0^;tim-gal4/* + *;uas-per16/* + males that express PER in all clock neurons. As negative controls, we used flies that do not express PER, i.e., *per^0^;Mai179-gal4/*+*;* + */* +, *per^0^;tim-gal4/* +*;* + */* +, and *per^0^;*+*;uas-per16/* +.

### Behavioral Experiments and Analysis

**FIGURE 2:**
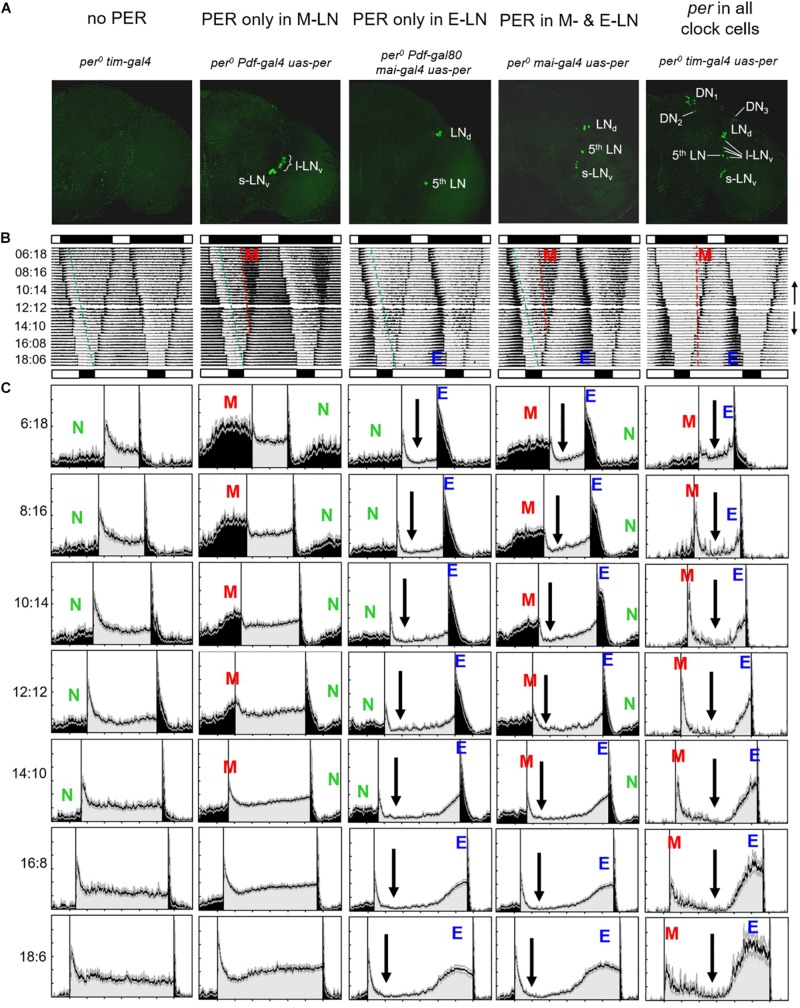
Average actograms and activity profiles of 25–30 flies, respectively, of the following lines: (1) *per*^0^ *tim-gal4* mutant controls (no PER), (2) flies with PER only in the eight PDF-positive lateral neurons (PER only in M-LN), (3) flies with PER only in the ITP-positive and sNPF-positive lateral neurons (per only in E-LN), (4) flies with PER in most lateral neurons (*per* in M- and E-LNs) and flies with PER in all clock neurons. **(A)** Representative anti-PER staining for the right brain hemisphere of each genotype, respectively. **(B)** Double plots of average actograms of all fly strains. The flies were entrained to subsequent LD cycles in which day length was either stepwise reduced (LD 12:12, 10:14, 8:16, and 6:18) or in which day length was stepwise increased (LD 12:12, 14:10, 16:8, and 6:18). Black and white bars above and below each actogram indicate the LD cycle of the shortest and longest photoperiod, respectively. To see the overall entrainment pattern, the average actograms of the short days and long days were combined in one composite actogram for each genotype, in which the short-day actogram (upper part of the composed actograms) was vertically flipped (see small arrows on the right margin indicating the directional flow of the actograms). These actograms give a rough comparison of the behavior of the different genotypes. Morning (M) and evening (E) activity bouts are visible in flies with PER in all clock cells as well as in M- and (E-LN-oscillator flies (see red and blue letters). Only an M activity bout is present in flies with PER only in the M-LN, and only an E activity bout is present in flies with PER only in the E-LN. Such activity bouts are absent in flies without PER. Under short days, *per*^0^ mutants show a considerably amount of nocturnal activity that starts a few hours after lights-off. The onset of this nocturnal activity is visible in all fly strains except the ones with per in all clock neurons and is marked by stippled green lines. The onset of M activity, which is only visible in flies with functional M oscillators and occurs clearly before lights-on, is marked by red stippled lines. **(C)** Average activity profiles for each strain under all photoperiods (the LD cycles are indicated at the left margin; activity during the light phase of the relevant LD cycle is shown in gray and activity during the dark phase is shown in black). The activity profiles are normalized in such a way that the highest activity was set to a value of 1. Colored letters mark nocturnal (N), morning (M), and evening (E) activity, respectively. Black arrows indicate reductions in activity level during the day.)

All flies were raised on cornmeal/agar medium supplemented with yeast at 20°C in LD12:12. At the age of 1–3 days, individual male flies were transferred into the recording chambers. Locomotor activity was recorded for 3 days under a LD12:12 cycle with the same phase as during rearing and a light intensity of 100 lux. Subsequently, the duration of the photoperiod was either increased to 14 h or reduced to 10 h for half of the animals, respectively (see Figure 2B). After 6 days of recording, the photoperiod was further increased (to 16 h) or reduced (to 8 h). This was repeated after further 6 days of recording, so that photoperiod was finally 18 or 6 h, respectively. Thus, the flies’ activity was recorded under the following LDs: (1) 12:12, 14:10, 16:8, 18:6, or (2) 12:12, 10:14, 8:16, 6:18. Throughout the whole recording period, data were collected every minute (i.e., in 1 min bins).

The raw data were displayed as actograms using the program actogramJ (Schmid et al., 2011). Flies that did not survive the first two light regimes were excluded from the analysis. From the others, average actograms and average activity profiles were calculated as described previously (Schlichting and Helfrich-Förster, 2015). For calculating average activity profiles, first, an average day was calculated for each fly including the second to the last day under each condition. The average days of individual flies were then used to calculate the average activity profiles for each fly group. We normalized the average activity profiles so that the maximal activity was 1. To reveal the phase of the beginning and maximum of morning (M) and evening (E) activity bouts, respectively, the raw data were smoothed with a moving average filter of 51 (i.e., each recorded bin is plotted as the average activity levels of 51 bins in total: the bin of interest plus 25 bins that precede and the 25 follow the bin of interest). This degree of smoothing made the activity profile more compact. The relevant times of M and E bouts could then be determined by manually selecting the starts and maxima with the mouse pointer. Mean phases with respect to midnight were calculated for each genotype under each photoperiod. The phase relationship between M and E peak (Y_M,E_) was determined for each fly and averaged for the different genotypes and photoperiods. In addition, we calculated the absolute average activity (beam crosses/10 min) for each fly during the entire 24 h day (= overall activity level), during the light phase (= diurnal activity), and during the dark phase (= nocturnal activity).

### Statistics

Activity levels and Ψ_M,E_ were tested for significant influence of photoperiod and genotype (different strains) using two-way analysis of variance (ANOVA) after testing the data for normal distribution with the Kolmogorov–Smirnov test (Systat 13 Version 13.00.05; SPSS, Chicago, IL). A Bonferroni *post hoc* test was applied for pairwise comparisons. Values were regarded as significantly different at *p* < 0.05 and as highly significantly different at *p* < 0.001. When data were non-normally distributed, *p*-values were adjusted through multiplication by 10, according to Glaser (1978).

## Results

### General Activity Patterns

#### *per*^0^ Controls

**FIGURE 3:**
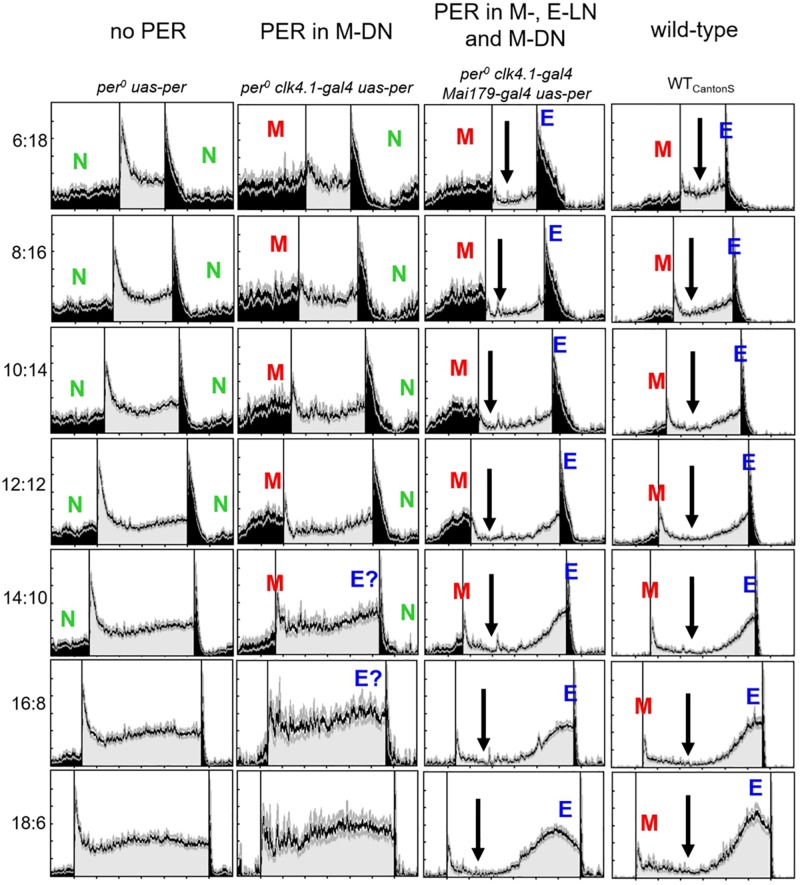
Normalized average activity profiles of 25–30 flies, respectively, of the following lines: (1) *per*^0^ mutant controls (no *per*), (2) flies with *per* only in ∼8 CRY-positive dorsal posterior neurons (*per* in DN_1__p_), (3) flies with *per* in most lateral neurons and in the DN_1__p_ (*per* in M- and E-LNs and DN_1__p_), and (4) wild-type flies (WT_CantonS_). Labeling as in Figure 1C.

All flies that lacked PER completely exhibited the same behavior, which is shown for “*per^0^ tim-gal4*” controls (*per^0^;tim-gal4/* +*;uas-per16/* +) in Figures 2B,C (1st column) and for “*per^0^ uas-per*” controls (*per^0^;* +*;uas-per16/* +) in Figure 3 (1st column). Under all tested photoperiods, the flies responded with high activity to lights-on and lights-off (the so-called lights-on and lights-off peaks), but they exhibited neither morning nor evening activity bouts and were more active during the day than during the night. After the lights-on peak, their diurnal activity level remained rather constant, whereas their nocturnal activity level dropped temporarily after the lights-off peak. This activity drop was more pronounced and lasted for the entire night under long photoperiods (light phase > 14 h), probably because the flies had been active throughout the entire long light phase and therefore physically exhausted (see average actograms in Figure 2B). The green stippled line in the average actogram (Figure 2B, 1st column) indicates the increase of nocturnal activity (as determined by visual inspection) after the initial drop. The latter is also visible in the average activity profiles and is marked by a green “N” in Figures 2, 3. The increase in nocturnal activity was slightly larger in *per^0^ uas-per* controls than in *per^0^ tim-gal4* controls (compare first columns in Figure 2 with that of Figure 3). Nevertheless, the absolute amount of nocturnal activity was the same in both *per*^0^ control strains (see later). In summary, the daily changes in activity of the *per*^0^ controls can be explained as responses to the external LD cycles and to their internal sleep need.

#### *per*^0^ Mutants With PER Rescued in All Clock Cells and Wild-Type Controls

The activity pattern of *per*^0^ mutants, in which PER was rescued under control of the *timeless* promotor in all clock cells (“*per^0^ tim-gal4 uas-per*” = *per^0^;tim-gal4/* +*;uas-per16/* +), was indistinguishable from that of wild-type flies (compare last row of Figure 2 with that of Figure 3). Both strains showed the typical activity pattern with M activity bouts around lights-on and E activity around lights-off under short and medium photoperiods. Only under long photoperiods (light phase > 16 h) did E activity peaks occur before lights-off. Between M and E activity, the flies held a siesta, which was longer under long photoperiods (arrows in Figures 2, 3). The activity pattern of *per^0^ tim-gal4 uas-per* (Figure 2C, last column) showed strong similarity to that of wild-type flies (Figure 3, last column). This suggests that the expression pattern of PER plays a more critical role in shaping locomotor activity than PER expression levels, which might differ between the transgenic *per^0^ tim-gal4 uas-per* line and wild types.

#### Flies With PER Only in the M-LN

In contrast to *per*^0^ controls, the flies with PER in the M-LN (“*per^0^ Pdf-gal4 uas-per*” = *per^0^;Pdf-gal4/* +;*uas-per16/* +) exhibited a pronounced M activity bout that occurred before lights-on under short photoperiods (Figure 2, 2nd column). Under LD12:12, M activity was still visible; it started before lights-on and reached peak levels around lights-on. However, under long photoperiods, morning activity and the lights-on peak were barely distinguishable. Under LD16:08, the “lights-on peak” of M-LN flies lasted still longer than that of *per*^0^ controls, but under LD18:06, the activity pattern of M-LN flies and *per*^0^ controls was very similar. Most importantly, M-LN flies showed no siesta and no E activity bout. Their diurnal activity level remained constantly high throughout the light phase without any activity increase in anticipation of lights-off (Figure 2, 2nd column). As already observed in *per*^0^ controls, lights-off provoked a lights-off peak after which nocturnal activity dropped almost to zero (see average actogram in Figure 2B, 2nd column). This activity drop lasted for about 2–3 h and then the nocturnal activity level visibly increased (green line in the average actogram and green “N” in the activity profiles). The M activity bout then constituted a second strong increase in nocturnal activity (red stippled line in the average actogram and red “M” in the activity profiles).

#### Flies With PER Only in the E-LN

Flies with PER in the E-LN (“*per^0^ Pdf-gal80 mai-gal4 uas-per*” = *per^0^;Mai179-gal4/Pdf-gal80;uas-per16/* +) exhibited no morning activity bout. Their nocturnal activity was very similar to that of the *per*^0^ controls (see stippled green line in the average actogram and the “N” in the activity profiles shown in Figure 2, 3rd column). However, the E-LN flies exhibited a less pronounced lights-on peak as compared to *per*^0^ mutants and their diurnal activity level after the lights-on peak dropped to very low levels (arrows in Figure 2B, 3rd column). Under short photoperiods, activity increased only after lights-off and manifested itself as a long and pronounced lights-off peak. Under long photoperiods, however, the E-LN flies showed a pronounced evening bout of activity that seemed to occur at about the same phase as it did in control flies with PER rescued in all clock cells (Figure 2B, 3rd column).

#### Flies With PER in the M- and E-LN

The activity pattern of flies with PER in M- and E-LN can be regarded as a mixture of that of flies with only M-LN or only E-LN (Figure 2, 4th column). The flies exhibited M and E activity bouts with a very similar phase to the flies with only one of the two LN oscillators. Most importantly, under short photoperiods, the phases of the two were not wild type like, but occurred earlier and later, respectively. After lights-on, the diurnal activity level dropped and remained low until the increase of evening activity (arrows in Figure 2B, 4th column).

#### Flies With PER Only in the M-DN

The activity pattern of flies with PER only in the M-DN was in principle similar to that of the M-LN flies. However, M activity was lower and the M activity bout was less defined (Figure 3, 2nd column), suggesting that the M-LN are needed for a pronounced M activity bout. Furthermore, the flies showed signs of E activity (marked as “E?” in Figure 3), suggesting that the CRY-positive DN_1__p_ cells that express PER in this line may also include some E oscillators.

#### Flies With PER in the M-, E-LN, and M-DN

The activity pattern of flies with PER in M-LN and M-DN plus E-LN was very similar to that of flies with PER in M-LN and E-LN, suggesting that PER in the M-DN does only marginally contribute to the appearance and phase of M and E activity (Figure 3B, 3rd column). Nevertheless, nocturnal activity (green “N” in Figure 2) appeared considerably lower in flies that had PER additionally in the M-DN. Diurnal activity dropped after M activity and remained low until the increase in E activity (arrows in Figure 3).

### Phases of M and E Activity Bouts and Phase Relationship Between the Two

**FIGURE 4:**
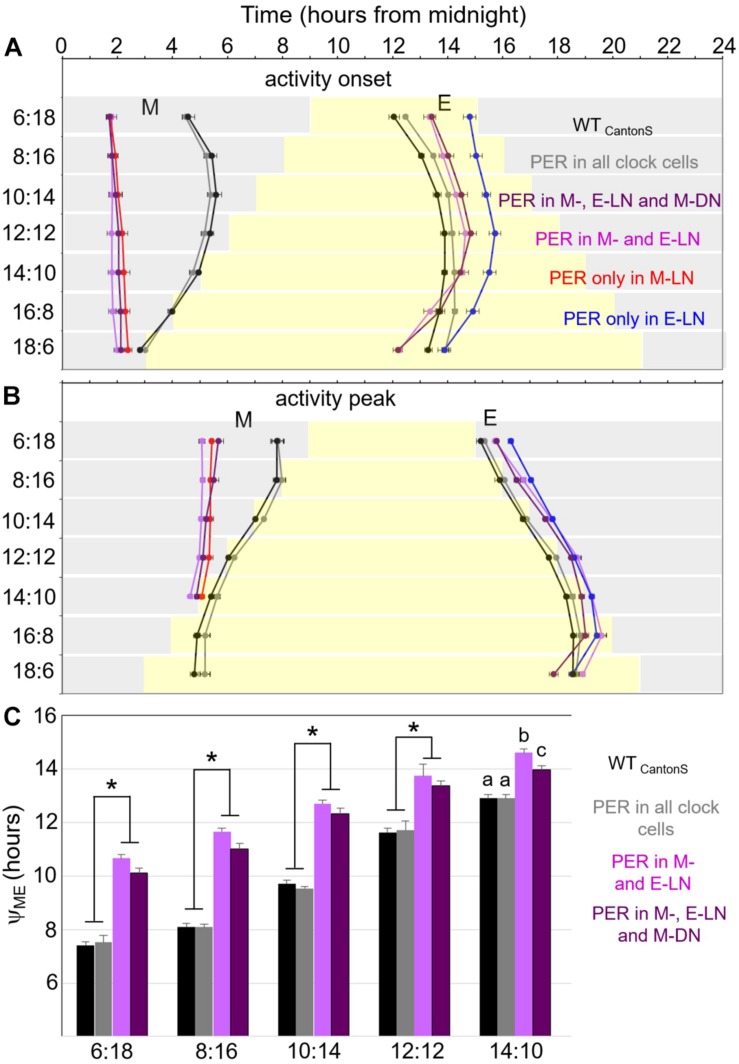
Timing of morning and evening activity in most of the strains shown in Figures 1, 2 under the different photoperiods. We were not able to reliably determine the phases of M activity in flies with PER only in the M-DN. Therefore, these flies are not included here. **(A)** Timing of onset of morning and evening activity, respectively. **(B)** Timing of morning and evening activity peaks. **(C)** Distances between morning and evening activity peaks (Y_ME_) until a photoperiod of 14 h. Error bars show standard errors of the mean. A two-way ANOVA revealed a clear dependence of Y_ME_ on photoperiod [*F*_(5, 604)_ = 486.579; *p* < 0.001] and on the strain [*F*_(3, 604)_ = 173.384; *p* < 0.001], as well as a significant interaction between both [*F*_(15, 604)_ = 7.204; *p* < 0.001], which indicates that the response to photoperiod is significantly different in the different strains. A *post hoc* analysis showed that Y_ME_ is significantly shorter in the wild-type strain (WT_CantonS_) and flies with PER rescued in all clock cells as compared to flies in which PER was only rescued in specific subgroups (*p* < 0.001, asterisks). At all photoperiods, there is a tendency that Y_ME_ is shorter in the flies, in which PER is rescued in the M-DN in addition to the M- and E-LN as compared to only in the M- and E-LN. However, this difference turned out to be only significant (*p* = 0.018) under the longest day (14:10) as indicated by different letters.

Since the precise phases of M and E activity bouts are hard to see in the average activity profiles, we determined the onset of M and E activity and their relevant peak phases for each fly and calculated the means for each fly strain (Figure 4). These plots show that only flies that expressed PER in all clock cells showed a wild-type phase of M and E activity. Flies with PER only present in the M-LN, in the M-LN and E-LN, or in the M-LN, E-LN, and M-DN had a very early onset of M activity as well as an early activity peak. E activity started the latest in flies with PER only in the E-LN, significantly earlier in flies with PER in the M-LN and E-LN or in the M-LN, E-LN, and M-DN, and the earliest in wild-type flies followed by flies with PER in all clock cells (Figure 4A). Interestingly, the slope of activity onset in dependence of photoperiod was different for the flies that expressed PER only in the subset of the clock neurons and those that expressed it in all cells. In flies with PER in all clock cells, E activity onset became later with increasing photoperiod, while in those that had per only in subsets of clock cells, this was only the case until a photoperiod of 12 h. With longer photoperiods (14, 16, 18 h), the phases of E onsets were advancing again, finally becoming earlier than the phase of E onset in wild-type flies (Figure 4A). In respect of M and E activity peaks, the difference between flies that express PER in all cells and flies that do so only in a subset of clock neurons was less dramatic, but otherwise similar: The M activity peak was significantly earlier in the flies with PER in a subset of clock neurons until a photoperiod of 16 h (Figure 4B). At longer photoperiods, we could not distinguish the M peak from the lights-on peak, and therefore, we could not determine its phase. The E activity peak occurred after lights-off in the flies that expressed PER only in subsets of the clock neurons, while it was around lights-off in wild-type flies. As observed for the onset of E activity, at long photoperiods, the E peak became earlier in flies with PER only in subsets of the clock neurons than that of wild-type flies (Figure 4B).

As observed in earlier studies (Rieger et al., 2012), the distance (phase relationship Y_ME_) between M and E peaks was small in wild-type flies under short photoperiods and lengthened considerably with increasing photoperiod (Figure 4C). The same was true for flies with PER in all clock cells. The other two strains, in which we could calculate Y_ME_ (flies with PER in M- and E-LN and flies with PER in M-, E-LN, and M-DN) had a significantly larger Y_ME_ at short photoperiods and Y_ME_ lengthened less dramatically with increasing photoperiod (Figure 4C). Y_ME_ was always longer in the flies that expressed PER only in the M- and E-LN than in those that possessed PER additionally in the M-LN. In summary, this indicates that Y_ME_ is the shorter the more clock cells express PER.

### Nocturnal Activity Bouts

**FIGURE 5:**
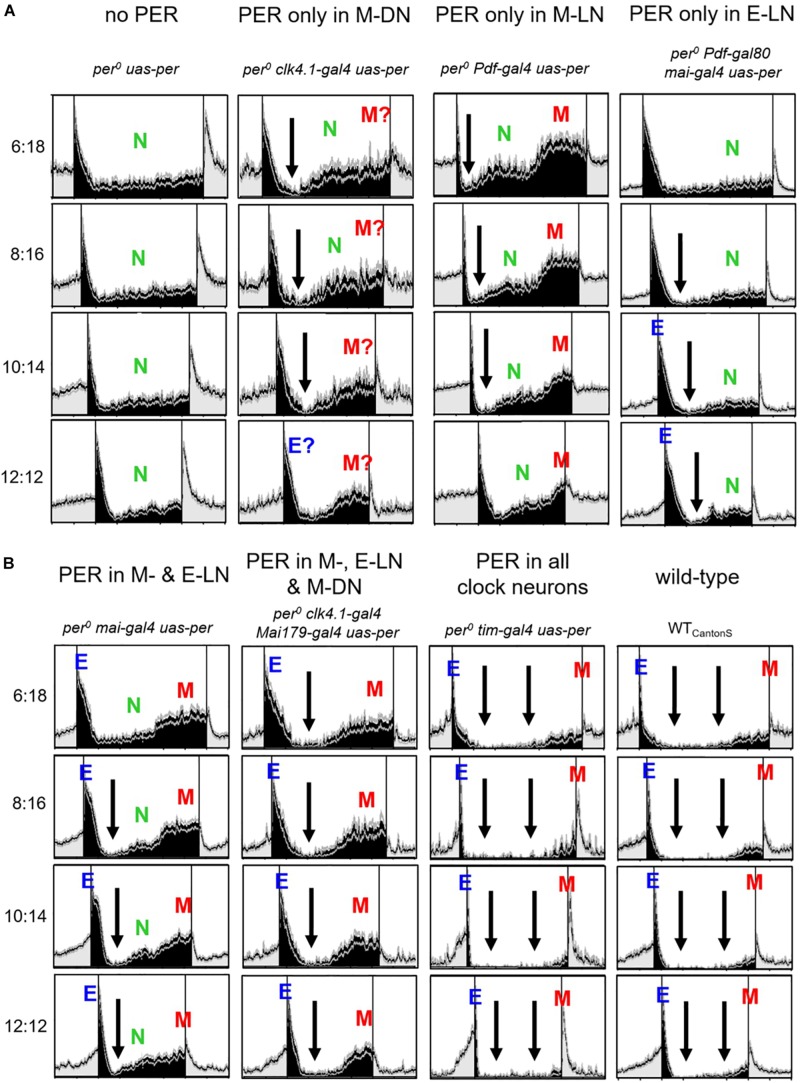
Nocturnal activity bouts of the different strains at short photoperiods (photoperiods between 6 and 12 h). The same normalized activity profiles shown in Figures 1, 2 are plotted in a way that the night is centered. In **(A)** are shown *per^0^ uas-per*; *per^0^ clk4.1-gal4 uas-per*; *per^0^ clk4.1-gal4 uas-per*; *per^0^ Pdf-gal4 uas-per*; and *per^0^ Pdf-gal80 mai-gal4 uas-per*; whereas in **(B)**, *per^0^ mai-gal4 uas-per*; *per^0^ clk4.1-gal4 mai-gal4 uas-per*; *per^0^ tim-gal4 uas-per*; and WT_CantonS_ are shown. Black arrows indicate times at which nocturnal activity is reduced. Otherwise, the labeling is similar to Figures 1, 2. Flies with PER in the M-DN show a lower M activity than flies with PER in the M-LN. Therefore, in these flies, it is more difficult to distinguish M activity from general nocturnal (N) activity and we added a question mark to the M peak. In flies with PER in M-, E-LN, and M-DN, N activity appeared absent and only the early M peak was visible, whereas in wild-type flies, N and M activity appeared largely suppressed. The latter is indicated by two black arrowheads.

One striking result of our study is the high nocturnal activity of most strains that express PER only in subsets of the clock cells under short photoperiods. To visualize nocturnal activity in more detail, we plotted the same normalized activity profiles shown in Figures 2, 3 once again, but this time with the night centered and only for the short photoperiods (Figure 5). While wild-type flies and flies with PER in all clock cells exhibited only minimal nocturnal activity, the nocturnal activity of all other strains was clearly higher. First, this indicates that a fully functional clock with PER present in all clock cells inhibits nocturnal activity (two large arrows in Figure 5, 3rd and 4th columns, respectively). In *per*^0^ controls, nocturnal activity (N) occurred at moderate constant levels throughout the night (Figure 5A, 1st column). This was comparable in flies with PER only in the E-LN (Figure 5A, 4th column), but here, N activity was lower and appeared already inhibited directly after E activity, as soon as the days get longer (>8 h, black arrows). In the other strains, activity was clearly inhibited directly after E activity (black arrows in Figure 5) or, in the case of flies with PER only in the M-LN (Figure 5A, 3rd column), directly after the lights-off peak, and this happened already under short photoperiods. After this drop, activity increased in two steps that can be best seen in flies with PER only in the M-LN or in the M- and E-LN (indicated as green “N” and red “M” in Figure 5A, 3rd column, and Figure 5B, 1st column, respectively). We interpret the second increase in activity as early M peak. In flies, in which PER is only present in the M-DN (Figure 5A, 2nd column), the second step in nocturnal activity increase is barely visible, suggesting that the M-DN activate the M peak only slightly so that it is hard to distinguish from the N peak. Nevertheless, when compared to *per*^0^ (Figure 5A, 1st column) controls, nocturnal activity is clearly enhanced when PER is present in the M-DN (Figure 5A, 2nd column). In flies expressing PER only in M-LN, E-LN, and M-DN (Figure 5B, 2nd column), the nocturnal activity is strongly suppressed, suggesting an inhibitory effect of the E-LN. Together, these findings indicate that some clock neurons inhibit nocturnal activity at certain times during the night while others promote it at other times of the night.

### Absolute Diurnal and Nocturnal Activity Levels

**FIGURE 6:**
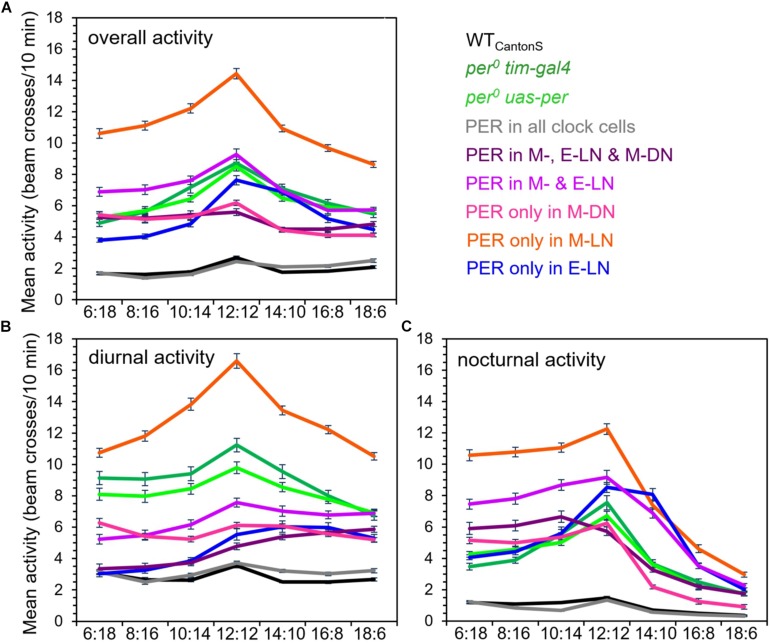
Absolute overall **(A)**, diurnal **(B)**, and nocturnal **(C)** activity levels in the different fly strains at all photoperiods (± SD). The activity values for the different strains are shown in different colors [color codes are given right to **(A)**]. A two-way ANOVA revealed a clear dependence of overall activity, on photoperiod [*F*_(7, 2_,_209)_ = 42.997; *p* < 0.001] and on strain [*F*_(8, 2_,_209)_ = 150.759; *p* < 0.001], as well as a significant interaction between both [*F*_(56, 2_,_209)_ = 3.677; *p* < 0.001]. Furthermore, two-way ANOVA revealed a clear dependence of diurnal and nocturnal activity on photoperiod [for diurnal activity, *F*_(7, 2_,_209)_ = 14.046; *p* < 0.001; for nocturnal activity, *F*_(7, 2_,_209)_ = 180.902; *p* < 0.001] and on the strain [for diurnal activity, *F*_(8, 1_,_109)_ = 125.203; *p* < 0.001; for nocturnal activity, *F*_(8, 2_,_209)_ = 107.405; *p* < 0.001], as well as a significant interaction between both [for diurnal activity, *F*_(56, 2_,_209)_ = 3.957; *p* < 0.001; for nocturnal activity, *F*_(56, 2_,_209)_ = 9.585; *p* < 0.001]. Together, this indicates that the responses of diurnal and nocturnal activity to photoperiod are significantly different in the different strains. No differences in overall, diurnal, and nocturnal activity levels were found between the *per*^0^ and wild-type controls, respectively. For more detailed explanations, see text.

So far, we have considered the relative activity of the flies throughout day and night on the normalized activity profiles. In order to see the effects of PER in the different clock neurons on real activity levels, we calculated the absolute average values of overall, diurnal, and nocturnal activity (in beam crosses per 10 min) at the different photoperiods (Figure 6). We found that, in most fly strains, the overall activity level was maximal at equinox (LD12:12) (Figure 6A). Nevertheless, the activity level was very different in the different strains. The highest overall activity level was found in flies that expressed PER only in the M-LN, while the lowest activity was present in wild-type flies. Arrhythmic *per*^0^ mutants showed intermediate activity levels and the activity of flies with PER rescued in different clock neurons clustered around that of the *per*^0^ mutants, sometimes below, sometimes above it (Figure 6A). These activity differences are consistent with the hypothesis that certain clock neurons promote activity (e.g., the M-LN), while other clock neurons rather inhibit activity (e.g., the E-LN and M-DN).

To get more insight into strain-dependent and photoperiod-dependent effects on diurnal and nocturnal activity, we calculated diurnal and nocturnal activity of the different strains at all photoperiods (Figures 6B,C). With few exceptions, diurnal activity was higher during the day than during the night. Deviations from this general pattern were found in flies that possessed PER in the M- and E-LN only or additionally in the M-DN. At short photoperiods, these flies were clearly more active during the night than during the day. Flies with PER only in the E-LN were also more active during the night than during the day but only under LD12:12 and 14:10.

As already found for overall activity, the highest diurnal and nocturnal activity levels of most strains occurred during equinox (LD12:12), respectively (compare Figures 6B,C). Diurnal activity decreased in most strains when the days became shorter or longer (Figure 6B), while nocturnal activity dropped only moderately under short days but prominently under long days (Figure 6C). Again, some strains differed from this pattern. For example, diurnal activity of flies with PER only in the E-LN or in the M- and E-LN remained high even under long days. Diurnal activity of flies with PER only in M-DN remained almost the same under all photoperiods (with a slight increase under very short days) and diurnal activity of flies with PER in the M-, E-LN, and M-DN steadily increased with photoperiod. Nocturnal activity of the latter flies remained constantly high under short photoperiods (until LD10:14) and then steadily decreased.

In sum, our analysis of the activity profiles and activity levels revealed that activity promotion and activity inhibition appear to happen in a time-dependent and photoperiod-dependent manner (see also Figures 2, 3, 5).

## Discussion

Since the discovery that specific clock neurons control M and E activity of *D. melanogaster*, many studies tested the properties of M and E oscillators in detail. In the following, we will discuss these studies in the light of our findings.

Most studies manipulated either the light sensitivity or the oscillation speed in the M and E clock neurons and tested the consequences on entrainment or free-running behavior of the flies (Stoleru et al., 2005, 2007; Murad et al., 2007; Picot et al., 2007; Zhang L. et al., 2010; Zhang Y. et al., 2010). They found that M and E oscillators do not only differ in their responsiveness to light as originally proposed by Pittendrigh and Daan (1976), but that they have in addition different capabilities to control rhythmicity under darkness and light.

Stoleru et al. (2007) expressed the kinase Shaggy in either the M cells or the E cells, which speeds up the clock in the relevant neurons, and recorded the flies’ activity under short and long photoperiods. They found that the M neurons controlled the phase of M and E activity phase under short days (both became early), while the E cells had no influence on the phase of M activity under these conditions. However, under long days, the situation was the other way around. Now, the E cells determined the phase of M and E activity, while the M neurons did not influence the phase of E activity. Stoleru et al. (2007) concluded that the M cells dominate on long nights and the E cells dominate on long days. Although the definition of E cells was not very accurate in this study (all clock cells except those of the M-LN were regarded as E cells), this result is very interesting. It fits our observation that flies with PER only in the M-LN drive a strong M activity bout in darkness under short days, but that this diminishes under long days. Vice versa, in flies with PER only in the E-LN, E activity is best visible in the light phase of long days. The differential dominance of M and E oscillators may even be reflected in the amplitudes of M and E peaks in wild-type flies under short and long photoperiods (Rieger et al., 2003) and in the flies with PER rescued in all cells shown in Figure 1. Under short days (LD 8:16 and 10:14), their M peak was higher than their E peak, whereas the opposite was true under long days (LD 16:8 and 18:6).

Other studies used the same definition of M and E neurons as we did in the present study, but they did not record activity under different photoperiods, but instead varied light intensity at equinox (LD 12:12) or recorded the flies under constant darkness (DD) or constant light (LL) (Picot et al., 2007; Rieger et al., 2009; Zhang Y. et al., 2010; Chatterjee et al., 2018). These studies found that functional M-LN neurons alone can control rhythmic behavior in constant darkness (DD), while functional M-DN or E-LN neurons alone cannot. Vice versa, the E-LN neurons alone can control rhythmicity in constant light (LL), but the M-LN or the M-DN alone cannot. Thus, the M neurons (especially the M-LN) appear to be dominant under DD, while the E neurons appear dominant under LL (reviewed in Yoshii et al., 2012), which fits the studies of Stoleru et al. (2007) and our observations. Picot et al. (2007) suggested the existence of a light-dependent switch between M and E oscillators that requires the visual system and helps the animal to adapt to seasonal changes in day length, but unfortunately, they have not recorded the flies under different photoperiods to test their hypothesis.

Finally, Chatterjee et al. (2018) found that M-LN are master oscillators that signal to the M-DN and both together control M activity. In other words, a functional clock is necessary in the M-LN and M-DN to control M activity in a wild-type manner. Indeed, we found here that flies that possessed PER in the M-LN and M-DN (in addition to the E-LN) had a more defined M activity under short days (they lacked the nocturnal activity peak “N”; see Figure 5). In addition, they had a more wild-type-like Y_M,E_ (Figure 4C) as compared to flies that possessed PER only in the M- and E-LN.

Chatterjee et al. (2018) also found that the E-LN and E-DN control E activity in parallel at low light intensities (∼50 lux), but that at higher light intensities (∼1,000 lux), the activity of the E-DN is blocked and solely the E-LN controls E activity. Thus, there is a light-dependent switch in the neuronal network controlling E activity. Most importantly, the *Clk4.1M* driver that we used to manipulate the M-DN drives also in some E-DN (Zhang Y. et al., 2010; Chatterjee et al., 2018; Figure 1) and the light intensity of 100 lux that we have used to record the flies probably allows the E-DN to contribute to E activity. This may explain why we have seen signs of E activity in our flies with PER in the M-LN, especially under long photoperiods (Figure 3), but most of the E activity in our recordings appears to stem from the E-LN.

Nevertheless, the situation is even more complex, because the E neurons are not independent of the M-LN (= s-LN_v_), or better to say from PDF stemming from the s-LN_v_ and the l-LN_v_ (Stoleru et al., 2005; Shafer and Taghert, 2009; Yoshii et al., 2009; Guo et al., 2014; Seluzicki et al., 2014; Yao and Shafer, 2014; Liang et al., 2016, 2017; Schlichting et al., 2016; Menegazzi et al., 2017; Chatterjee et al., 2018). In brief, the E neurons express the PDF receptor and respond to PDF, secreted from either the s-LN_v_ or the l-LN_v_ neurons, with a delay of their rhythm in neuronal activity (visualized by cellular Ca^2+^ levels). Subsequently, this leads to a delay in E activity. Such a delay is especially important under long photoperiods in order to keep E activity close to dusk. Two studies show that under long photoperiods, PDF comes predominantly from the l-LN_v_ neurons (Menegazzi et al., 2017; Schlichting et al., 2019b), while it appears to originate mainly from the s-LN_v_ cells under short photoperiods and equinox (Guo et al., 2014). Once more, this indicates that there is a light-mediated circuit switching in the *Drosophila* neuronal clock network, when light conditions change. The neuronal activity of the l-LN_v_ is highly dependent on light (Cao and Nitabach, 2008; Shang et al., 2008; Sheeba et al., 2008), which fits their dominant role as E activity-delaying mediators under long photoperiods.

Finally, yet importantly, there is also evidence that the M-DN cells feedback on the E-LN (Guo et al., 2016, 2018) and M-LN (Hamasaka et al., 2007) and block their activity via glutamate signaling. This leads to a block in M and E activity during midday, provoking the flies’ siesta. Most interestingly, high light prolongs the siesta even under equinox conditions (Rieger et al., 2007), and this is provoked by a special high light intensity pathway that signals to the M-LN and then via PDF to the M-DN that in turn blocks the activity of M-LN and E-LN (Schlichting et al., 2019a). These studies have been performed only under equinox conditions; nevertheless, it is well imaginable that the described pathways are also valid under long photoperiods in which a blocking of M and E oscillators during midday is especially important to prolong the siesta and delay E activity.

In the present study, we found that wild-type flies exhibited very little activity during midday and the night, while the activity level of *per*^0^ mutants was significantly higher throughout the entire 24 h day; in particular, no siesta was visible during midday. Although we cannot exclude the idea that the genetic background of the strains used might play a role, our results indicate that a main function of the circadian clock is to inhibit activity at less favorable times of the day. This nicely coincides with the findings of Menegazzi et al. (2012) and Schlichting et al. (2015), who tested the function of PER under more natural-like conditions and found that PER was especially needed to prevent activity of the flies during midday and the night. The same may be true for mammals. Chipmunks [in which the clock in the suprachiasmatic nuclei (SCN) was lesioned] that were released into the wild spent significantly more time outside their burrow during the night and consequently had a higher predation risk as compared to control animals (DeCoursey et al., 2000).

Here, we show that a functional circadian clock restricted to the main neuronal clusters composing the M and E oscillators (M-LN, M-DN, and E-LN) is not sufficient to inhibit activity in a wild-type-like manner. We also show that the different clock neurons do not all inhibit activity, and if they do so, they inhibit it at different times of the day. For example, the M-LN did not inhibit activity at all, but instead strongly provoked it. This is especially true for nocturnal activity under short photoperiods, conditions under which the M-LN induce a prominent nocturnal M activity bout (Figures 5, 6). However, also diurnal activity of the M-LN was rather high. No siesta occurred, and this was valid under all photoperiods. In contrast to the M-LN, the M-DN clearly inhibited activity, especially diurnal activity (Figure 6), which is in concordance with their siesta-inducing role. The absolute level of nocturnal activity was similar to that of *per*^0^ mutants, but as discussed above, the M-DN nonetheless suppressed nocturnal activity (N) after the lights-off peak (or the weak “E?” peak) (Figure 5). The highest activity inhibition during the day is caused by the E-LN, or by the E-LN in combination with the M-DN (as discussed above, the M-LN that also contain PER in the latter combination do not contribute at all to the inhibition of diurnal activity).

After having said this, the impression arises that nothing is left of the original Pittendrigh and Daan (1976) model, which explained the increasing Y_M,E_ by an acceleration of the M oscillators and slowing down of the E oscillators by light with increasing photoperiod. All appears explainable by provoking and inhibiting activity via different clock neurons at specific times of the day. However, this is only true at first glance. We found that the action of the different clock neurons on activity clearly depended on the photoperiod. Furthermore, Y_M,E_ also depended significantly on photoperiod and increased with increasing day length, even in the flies that expressed PER only in subsets of the clock neurons (Figure 4C). Thus, light has most likely different effects on the oscillation speed of M and E neurons. Indeed, flies with PER only in the M-LN, M-DN, and E-LN and that additionally lacked CRY showed an internal desynchronization into two components that free-run with short and long periods, respectively – a behavior that was similar to that of *cry*^0^ mutants with fully functional clock (Yoshii et al., 2012). Thus, in principle, the M oscillators appear to speed up and the E oscillators appear to slow down upon light, as predicted by Pittendrigh and Daan (1976). However, in addition to the oscillation speed, the dominance of *Drosophila’s* M and E oscillators changes with increasing light. This makes it hard to observe the velocity changes in M and E cells of flies that possess all sets of photoreceptors, including CRY. Furthermore, not all clock neurons behave as M and E oscillators (e.g., the l-LN_v_), and of several clock neurons, we still do not know the exact function (see Figure 1). Most likely, there are more than just M and E neurons and the so-called M and E neurons can adjust their function depending on the environmental conditions.

In summary, there is growing evidence that the circadian clock of *Drosophila* is composed of a plastic network of oscillators that rearrange themselves depending on the environmental demands. This explains why the original M and E oscillator model of Pittendrigh and Daan (1976) is too simple to describe the situation in *Drosophila*. Here, we show that PER expression within different subsets of clock neurons is not sufficient to adapt the behavior of the flies to different photoperiods in a wild-type manner (see Table 1). Perhaps PER is not only necessary in all clock neurons, but additionally in all glial cells for a wild-type behavior. Glial cells are still largely neglected in circadian studies, although several studies show that they play active roles in the clock (Zerr et al., 1990; Ewer et al., 1992; Ng et al., 2011; Jackson et al., 2015, 2019; Brancaccio et al., 2019). Future studies are warranted to test this important issue.

**TABLE 1:** Contribution of different clock neuron clusters in shaping the wild-type locomotor activity profile of *D. melanogaster*.

**Genotype**	**PER^+^ cells**	**Function/Phenotype short days**	**Function/Phenotype long days**
*per^0^ pdf-gal4 uas-per*	M-LN	Promote M activity	
*per^0^ pdf-gal80 mai-gal4 uas-per*	E-LN	Promote E activity	Promote E activity
		Inhibit day activity	Inhibit day activity
*per^0^ mai-gal4 uas-per*	M-LN, E-LN	Promote M activity	Promote E activity
		Promote E activity	Inhibit day activity
		Inhibit day activity	Partially inhibit E activity
*per^0^ clk4.1-gal4 uas-per*	M-DN	Promote M activity	Might promote E activity
*per^0^ mai-gal4 clk4.1-gal4 uas-per*	M-LN, E-LN, M-DN	Promote M activity	Promote E activity
		Promote E activity	Inhibit day activity
		Inhibit day activity	
		Inhibit N activity	
per^0^ tim-gal4 uas-per	All clock cells	M and E activity that track lights-on and -off	
		(reduced time difference between M and E)	
		Strong inhibition of day activity	
		(i.e., pronounced siesta)	
		Strong inhibition of night activity	

## Data Availability Statement

The raw data supporting the conclusions of this article will be made available by the authors, without undue reservation, to any qualified researcher.

## Author Contributions

PM, KB, VG, and MS performed the experiments. PM, KB, and CH-F conceived the study and planned the experiments. CH-F wrote the manuscript with contributions from PM, KB, and FS. All authors analyzed the experiments.

## Conflict of Interest

The authors declare that the research was conducted in the absence of any commercial or financial relationships that could be construed as a potential conflict of interest.
